# Supplementary material for: Content-rich biological network constructed by mining PubMed abstracts
Source: BMC Bioinformatics. 2004 Oct 8;5:147. doi: 10.1186/1471-2105-5-147 (PMC528731; doi:10.1186/1471-2105-5-147)
Supplement: Additional File 2 — The original results of the above study (non-essential files are deleted to keep the file size under the limit set by BMC bioinformatics). [file 1471-2105-5-147-S2.bz2 › chilibotAdditionalFile2/dip05/351ID8663118E4684/html/IL13_IL13RA2.html]

 


 **IL13** and **IL13RA2** 
  
Found 82 abstracts in PubMed, retrieved 05.  
 

 What does Google say? 
 PDF only 
| .edu only 

---

**Interactive relationship** (e.g. stimulation, inhibition, etc)

**Non-interactive relationship** (e.g. studied together, co-existance, homology, etc.)

- We have previously reported that interleukin 13  [ **IL13** ]  receptors IL 13R  [ **IL13RA2** ]  are constitutively overexpressed on a majority of human malignant glioma cell lines and primary cell cultures.  Ref: 12481422 Mol Cancer Ther, 2002
- This was followed by successfully treating xenograft tumor bearing nude mice with IL 13R  [ **IL13RA2** ]  directed cytotoxin  **IL13**  PE38QQR.  Ref: 12471149 J Immunol, 2002
- Interleukin 13  [ **IL13** ]  receptor IL 13R  [ **IL13RA2** ]  alpha2 chain plays a key role in ligand binding and internalization.  Ref: 12808442 Gene Ther, 2003
